# Influence of the Color, Shape, and Size of the Clay Model on Arthropod Interactions in Turfgrass

**DOI:** 10.1093/jisesa/ieab070

**Published:** 2021-10-20

**Authors:** Fawad Z A Khan, Shimat V Joseph

**Affiliations:** Department of Entomology, University of Georgia, 1109 Experiment Street, Griffin, GA 30223, USA

**Keywords:** biological control, predator–prey interactions, impressions

## Abstract

Many predatory arthropods occur naturally in turfgrass, and they provide adequate control of lepidopteran pests, such as fall armyworm, *Spodoptera frugiperda* (JE Smith) (Lepidoptera: Noctuidae), and black cutworm, *Agrotis ipsilon* (Hufnagel) (Lepidoptera: Noctuidae). Recording predation is challenging under field conditions because predators rarely leave any evidence. Clay models were successfully employed for studying predation, and this technique is underutilized in turfgrass. Little is known about whether the characteristics of clay models, such as color, shape, and size, influence arthropod interactions in turfgrass. To improve the utility of clay models in turfgrass, the influence of the color, shape, and size of clay models on arthropod interactions was studied by exposing clay models during daytime and nighttime in a turfgrass field. The results showed that arthropods interacted with clay models, and various types of impressions were recorded, including paired marks, scratches, cuts, and pricks. Although the color of the clay model had no significant effects on arthropod interactions during the night, significantly greater numbers of impressions were noticed on the blue and green models than on the yellow models during the daytime. The caterpillar-shaped models captured significantly greater densities of impressions than the beetle-shaped models. Additionally, the number of impressions significantly increased with an increase in the size of the model regardless of shape.

Turfgrass is a perennial grass that is regularly managed at low height as a uniform green ground cover (Robbins and Birkenholtz 2003, Held and Potter 2012), which essentially adds aesthetic, recreational, and environmental benefits to the landscape (Stier et al. 2015). Turfgrass is the largest cultivated crop in the United States, covering approximately 20.2 million ha (Milesi et al. 2009) and contributing $58 billion annually to the U.S. economy (Haydu et al. 2008). Turfgrass supports a diverse group of arthropod fauna, such as herbivores (Potter and Braman 1991, Eickhoff et al. 2006, Nair et al. 2021), pollinators (Del-Toro and Ribbons 2020, Joseph et al. 2020), predators (Braman et al. 2002, 2003; Joseph and Braman 2016), parasitoids (Braman et al. 2004, Joseph and Braman 2011), and detritivores (Joseph and Braman 2009a). Predatory arthropods, such as anthocorids, Araneae, carabids, formicids, geocorids, mirids, lasiochilids, and staphylinids, are abundant and common on turfgrass (Joseph and Braman 2009a, Singh 2020). These predators can control key turfgrass pests, such as fall armyworm, *Spodoptera frugiperda* (JE Smith) (Lepidoptera: Noctuidae) (Joseph and Braman 2009b), black cutworm, *Agrotis ipsilon* (Hufnagel) (Lepidoptera: Noctuidae) (López and Potter 2000), Japanese beetle, *Popillia japonica* Newman (Coleoptera: Scarabaeidae), billbugs, *Sphenophorus* spp. (Coleoptera: Curculionidae) (Dupuy and Ramirez 2019), and southern chinch bug, *Blissus insularis* Barber (Hemiptera: Blissidae) (Nachappa et al. 2006). Thus, to obtain sustained benefits from natural pest control in turfgrass systems, these predators should be conserved.

Predator–prey interactions can be studied through various techniques, such as direct observations (Pfannenstiel and Yeargan 2002, Cabrera et al. 2019), video recordings (Zou et al. 2017, Manubay and Powell 2020), caged experiments (Li et al. 2017), live sentinel prey baits (Tillman et al. 2020), quantitative fatty acids (Iverson et al. 2004), stable isotopes (Boecklen et al. 2011, Kamenova et al. 2018), DNA in gut content (Eitzinger et al. 2019, Oliveira-Hofman et al. 2020), and impressions on clay models (Bateman et al. 2017, Rößler et al. 2019, Khan and Joseph 2021). Among these techniques, the use of a clay model resembling insect prey is a cost-effective and emerging tool that could be utilized to estimate predation in various ecosystems (Howe et al. 2009, Lövei and Ferrante 2017, Rößler et al. 2018), including forest (Sam et al. 2015, Molleman et al. 2016, Gunnarsson et al. 2018, Hariraveendra et al. 2020), agricultural (Mansion-Vaquié et al. 2017, Denan et al. 2020), and urban ecosystems (Long and Frank 2020, Nason et al. 2021, Pena et al. 2021). To record predaceous activity, clay models simulating prey organisms are prepared and exposed to predators in the ecosystem. After the exposure, the clay models are recovered, and impressions created on the clay models are evaluated to estimate possible predatory interactions. Distinct impressions found on the clay model provide insights into understanding predator species and their activity and behavior (Low et al. 2014, Khan and Joseph 2021).

Invertebrate and vertebrate predators interact with clay models and create distinct impressions (Bateman et al. 2017, Lövei and Ferrante 2017, Khan and Joseph 2021). Arthropod predators are active in the ground, especially on the temperate forest floor (Ferrante et al. 2017). They use chemical, tactile, visual, and gustatory cues to search and locate prey (Yasuda 1997, Halpin and Rowe 2016, Duong et al. 2017, Xue et al. 2018, Yamazaki et al. 2020). Along with the color and color patterns, the ambient light availability, shape, and size of the insect also play a role in determining the visual perception of the predator (Troscianko et al. 2009). Previous studies showed that body size (Remmel and Tammaru 2009, Moura et al. 2018, Sahayaraj and Fernandez 2021), coloration (Théry and Gomez 2010, Zvereva et al. 2019, Aslam et al. 2020), and shape (Paluh et al. 2015) could influence the behavior of arthropod predators and how they interact with clay models. Additionally, predation rates can vary and could be subject to the difference in the appearance of prey or the reflectance of light from the model (Rojas et al. 2014, Cheng et al. 2018). However, the effects of the characteristics of the clay model, such as color, size, and shape, on arthropod predator interactions are not documented in turfgrass field settings. Moreover, the activity of arthropod predators and prey insects can vary during the daytime and nighttime hours. Thus, the objectives of the current study were to determine the effects of 1) color, 2) size, 3) shape of the clay model, and 4) time of exposure on predatory interactions in the turfgrass system.

## Materials and Methods

### Study Site and Clay Model

In 2020, experiments were conducted on ‘Tifway’ bermudagrass (*Cynodon* spp.) plot (2,896.4 m^2^) located at the University of Georgia, Griffin Campus, Griffin, GA (33.2622, −84.2829). The plot is part of a 71,890.5-m^2^ open turfgrass research field with no trees within 50 m from all directions. The bermudagrass was mowed weekly at 8 cm height and irrigated daily for 30 min. However, regular fertilizer and pesticide applications were not administered. Although the bermudagrass field was partially infested with weeds, treatments were deployed where bermudagrass was continuously present. All the experiments were conducted on the same turfgrass plot.

The clay models were prepared using nontoxic clay (Sculpey III, Polyform Products, Elk Grove Village, IL). This clay product was selected because it stays soft under field summer temperatures (Roels et al. 2018).

### Color and Time of Exposure

**Fig. 1.**
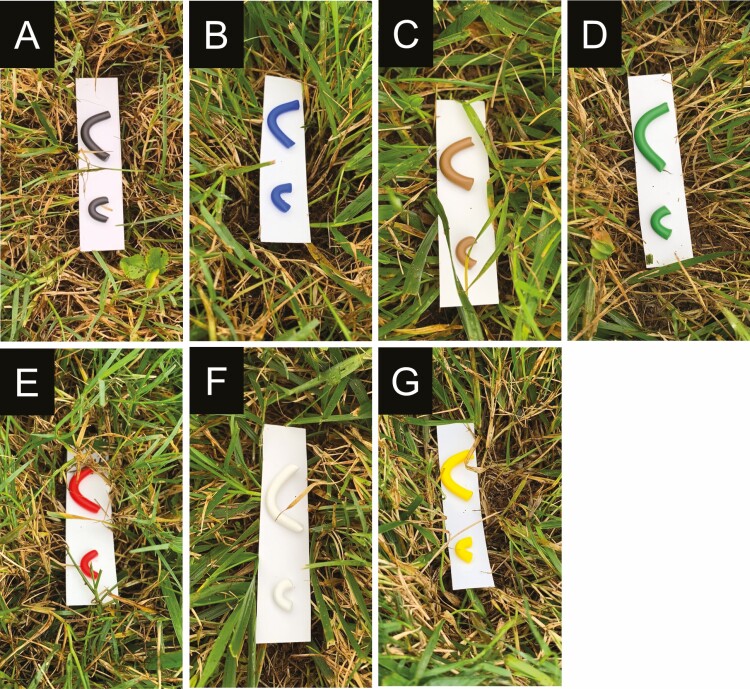
Clay models of different colors: (A) black-, (B) blue-, (C) brown-, (D) green-, (E) red-, (F) white-, and (G) yellow-colored models.

**Fig. 2.**
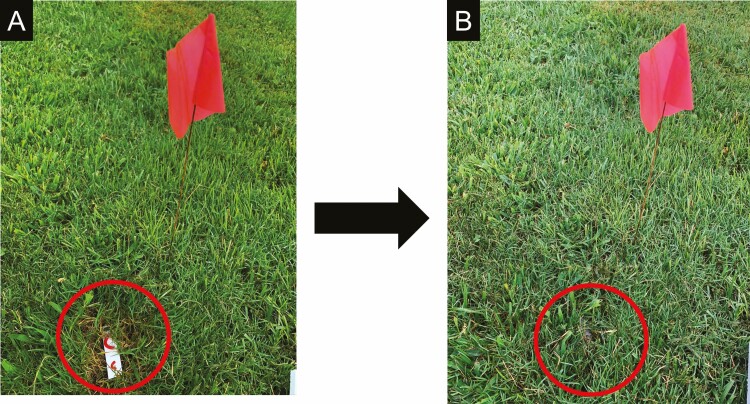
Method of placing the clay model experimental unit in turfgrass: (A) predeployment and (B) postdeployment.

Clay models were prepared using yellow- (Sculpey III yellow), blue- (Sculpey III blue), green- (Sculpey III string bean), black- (Sculpey III black), red- (Sculpey III red), white- (Sculpey III white), and brown- (Sculpey III hazelnut) colored clay (Fig. 1). The treatments included light and dark shades of colors. For each color, 10 × 2 mm (small) and 30 × 4 mm (large) (length × diameter) models were prepared to simulate early (third) and late (fifth) instars of *S. frugiperda* larvae, respectively. The treatments were seven colored clay models and time of exposure, daytime and nighttime hours. The colored models were deployed from 6:30 a.m. to 8:30 p.m. for daytime and from 8:30 p.m. to 6:30 a.m. for the nighttime. The clay models, a small and a large model, were glued on a 7.5 × 2 cm (length × width) weatherproof paper card (JL Darling, Tacoma, WA) using nontoxic glue (Newell Rubbermaid Inc., Westerville, OH), and it served as the experimental unit. The colored clay model treatments were arranged in a randomized complete block design (RCBD) with 6 or 10 replications, whereas the time of exposure treatment was replicated three times. The colored clay model treatments were deployed at 3-m spacing within a block and between blocks. The colored clay model treatments were deployed 6 m from the edge of the turfgrass field. The individually colored treatment was placed on the surface of the thatch after clearing the turfgrass canopy (Fig. 2). The experiment was repeated where colored clay model treatments were replicated 6 times in the first trial and replicated 10 times in the second trial. Trial 1 was conducted from 19 to 21 May, and trial 2 was conducted from 29 to 31 July 2020, representing the early and mid-summer months in Georgia.

### Shape and Size

**Fig. 3.**
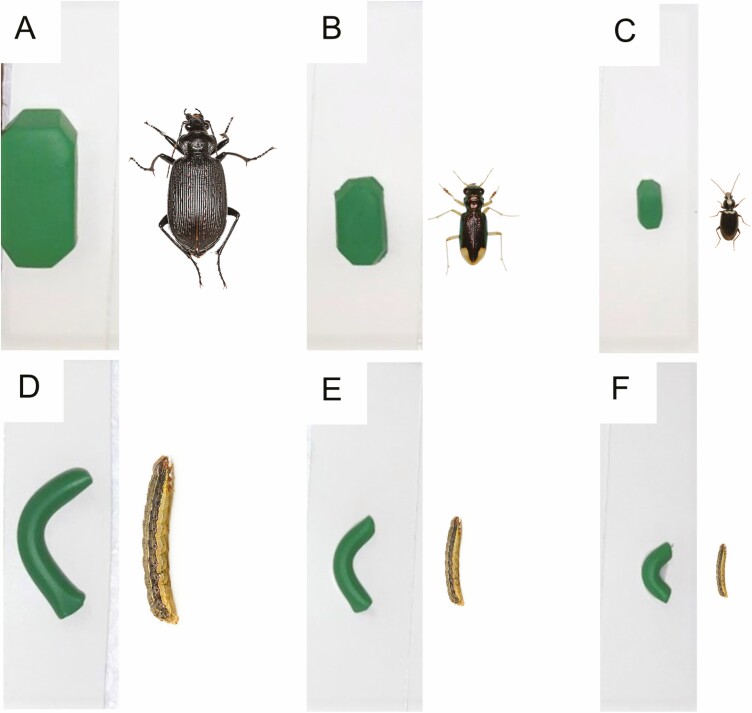
Clay models of different shapes and the corresponding insects: (A) large beetle model and *Calosoma sayi* Dejean (Coleoptera: Carabidae), (B) medium beetle model and *Tetracha carolina* (L.) (Coleoptera: Carabidae), (C) small beetle model and *Agonum* sp. (Coleoptera: Carabidae), (D) large larval model and fall armyworm, (E) medium larval model and fall armyworm, and (F) small larval model and fall armyworm.

For clay model preparation, the same procedure as described previously was adopted, but only green-colored clay was used. Previous studies showed that predators interacted with green-colored clay models (Low et al. 2014, Sam et al. 2015, Roels et al. 2018, Khan and Joseph 2021, Long and Frank 2020). Elongated cylindrical- and rectangular octagonal-shaped models were prepared for the experiment. The elongated cylindrical shape represented *S. frugiperda* larvae, whereas large, medium, and small shapes represented various stages of larvae. The three rectangular-octagonal shapes represented adults of predaceous carabids, *Calosoma sayi* Dejean, *Tetracha carolina* (L.), and *Agonum* spp., respectively (Fig. 3). The three sizes for *S. frugiperda* larvae were 30 mm × 5 mm, 17 mm × 3.5 mm, and 10 mm × 2.5 mm (length × diameter), whereas the predatory beetles were 26 mm × 12 mm × 8 mm (*C. sayi*), 14 mm × 8 mm × 6 mm (*T. carolina*), and 7 mm × 4 mm × 3.5 mm (*Agonum* spp.) (length × width × height). The models were individually glued on a 7.5 cm × 2 cm (length × width) weatherproof paper card using nontoxic glue.

Clay model treatments were deployed at 3-m spacing within a block and between blocks. To reduce the edge effect, the first block was 6 m away from the edge of the turfgrass field. The treatments were placed on the soil surface after mowing the turfgrass canopy and were exposed for 24 h, from one morning (10:00 a.m. to 12:00 p.m.) to the following day. The treatments in the experiment were clay model shape and size, and they were arranged in RCBD with 10 replications. The experiment was conducted twice, from 1 to 3 July and from 4 to 6 August 2020. The assays were replicated 10 times for each trial.

### Evaluation

Clay models were recovered from the field, transported to the laboratory, and stored at room temperature (21°C) until evaluation. The clay models were evaluated, referring to the impression types characterized by Khan and Joseph (2021) using a dissecting stereomicroscope (40×). The impression types were categorized as paired marks, scratches, detached segments, pricks, dents, and U-shaped marks. Some impressions, such as deep distortions, merged surfaces, and scooped marks, were quantified as a percentage of the affected clay model surface area. Additionally, the clay models were evaluated for damage scales from 0 to 10. The damage scale could be interpreted as 0 (0%), 1 (1–10%), 2 (11–20%), 3 (21–30%), 4 (31–40%), 5 (41–50%), 6 (51–60%), 7 (61–70%), 8 (71–80%), 9 (81–90%), and 10 (91–100% of the clay model surface covered with the impressions).

### Statistical Analyses

All the data analyses were performed in SAS (SAS Institute 2012). For the color experiment, the numbers of impressions on the clay model treatments were subjected to analysis of variance (ANOVA) using the PROC GLIMMIX procedure in SAS. The procedure used a generalized linear mixed model with a negative binomial distribution and log link function. The colored clay model, time of exposure, and their interaction were the treatments. The treatments served as a fixed effect, whereas replications (6 or 10) served as a random effect. The estimation method was maximum likelihood with the Laplace approximation. To understand the effects of clay model color, the impressions were further subjected to one-way ANOVA by time exposure using the PROC GLIMMIX procedure in SAS. The clay model color and replication were included in the generalized linear model. Because data were analyzed using a generalized linear model, the data were neither assessed for normality nor transformed. Pearson’s correlation analysis was performed between impression types and total impressions using the PROC CORR procedure in SAS. If correlations existed between impression types and total impression, multicollinearity was removed by adding a PARTIAL statement to the PROC CORR procedure.

For the shape experiments, the number of impressions on the clay models was subjected to ANOVA by a generalized linear mixed model with a negative binomial distribution and log link function using the PROC GLIMMIX procedure in SAS. The treatments, shape, and size of the clay model were the fixed effects, and replications served as a random effect. The estimation method was maximum likelihood with the Laplace approximation. To understand the effects of size, the impressions were further subjected to one-way ANOVA by shape using the PROC GLIMMIX procedure in SAS. The clay model size and replication were included in the generalized linear model. Pearson’s correlation analysis between impression types and total impressions at a 95% significance level was performed using the PROC CORR procedure in SAS. If a correlation existed between impression types and total impressions, the multicollinearity was removed by adding a PARTIAL statement to the PROC CORR procedure in SAS. The means and standard errors of the variables were calculated using the PROC MEANS procedure in SAS.

## Results

### Impression Types

**Fig. 4.**
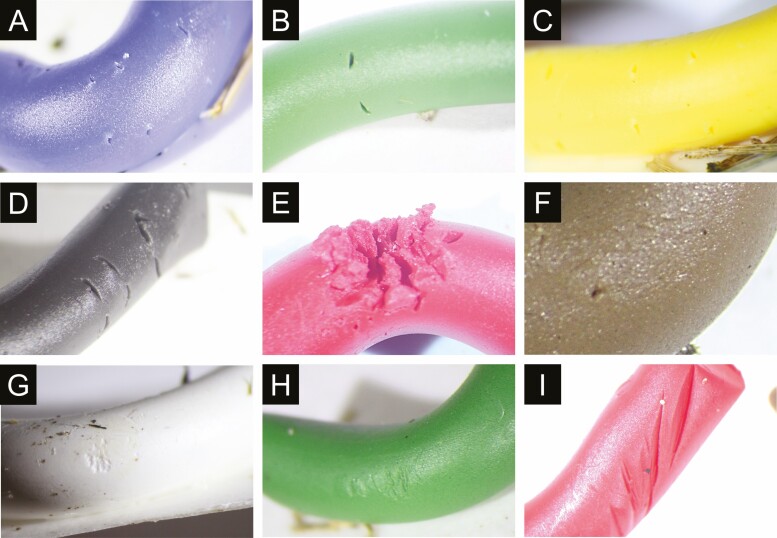
Impression types: (A–D) paired marks, (E) deep distortion, (F) pricks, (G) scooped marks, (H) scratches, and (I) cuts.

Ten impression types were observed during four trials in the field, and they were paired marks, scratches, cuts, detached segments, deep distortion, pricks, dents, merged surface impressions, scooped marks, and U-shaped impressions (Fig. 4). Of these impressions, paired marks, scratches, and cuts were most frequent. The less frequent impression types were summed up under the ‘other impressions’ category.

### Color and Time of Exposure

In trial 1 (May 2020), the clay model color and time of exposure had a significant effect on the total number of impressions, but the color × time of exposure interaction was not significantly different (Table 1). For the paired marks, the effects of model color and model color × time of exposure interaction were significantly different but not significantly different for the time of exposure. There was no significant effect of model color × time of exposure for cut, scratch, and other impressions (Table 1). When one-way ANOVA was performed by the time of exposure, none of the colors showed significant differences between each other for numbers of impression types and total impressions (Table 2).

**Table 1.** Summary of ANOVA results for the model used to find the effect of the color, exposure time, and their interaction on different impression types and their total number in May and July 2020

Impression	Exposure time			Color			Exposure time × color		
	F	df	P	F	df	P	F	df	P
May 2020									
Cut	0.0	1, 232	0.993	0.0	6, 232	1.000	0.0	6, 232	1.000
Paired	0.0	1, 232	0.950	2.1	6, 232	0.049	2.5	6, 232	0.022
Scratch	0.0	1, 232	0.992	1.5	6, 232	0.165	0.9	6, 232	0.473
Other	3.4	1, 232	0.065	0.9	6, 232	0.600	0.5	6, 232	0.832
Total	24.0	1, 232	<0.001	2.2	6, 232	0.047	2.1	6, 232	0.060
July 2020									
Cut	0.0	1, 397	0.986	0.4	6, 397	0.875	0.2	6, 397	0.990
Paired	21.7	1, 397	<0.001	1.3	6, 397	0.246	1.2	6, 397	0.323
Scratch	0.0	1, 397	0.999	6.1	6, 397	<0.001	1.4	6, 397	0.204
Other	0.6	1, 397	0.441	2.8	6, 397	0.010	1.1	6, 397	0.374
Total	5.0	1, 397	0.025	6.0	6, 397	<0.001	2.0	6, 397	0.069

**Table 2.** ANOVA and mean (± SE) number of impressions on various colors of clay models evaluated in May 2020

Exposure time		Impression type				
	Color	Paired mark	Scratch	Cut	Others^*a*^	Total
Daytime						
	Black	0.00 ± 0.00	0.11 ± 0.11	2.94 ± 1.81	0.33 ± 0.24	3.28 ± 1.91
	Blue	0.35 ± 0.35	0.18 ± 0.18	0.00 ± 0.00	0.41 ± 0.26	0.53 ± 0.38
	Brown	0.06 ± 0.06	0.33 ± 0.23	0.89 ± 0.68	0.33 ± 0.20	1.33 ± 0.76
	Green	1.28 ± 0.76	2.28 ± 1.43	0.11 ± 0.11	0.44 ± 0.22	3.83 ± 1.30
	Red	0.72 ± 0.43	2.28 ± 1.43	3.61 ± 2.45	0.72 ± 0.36	7.28 ± 4.12
	White	0.22 ± 0.13	1.06 ± 0.95	1.17 ± 1.05	0.33 ± 0.28	2.72 ± 2.33
	Yellow	0.17 ± 0.12	0.22 ± 0.22	1.33 ± 0.98	0.39 ± 0.16	1.83 ± 1.13
	*F*	1.3	1.9	1.6	0.2	1.2
	df	6, 113	6, 113	6, 113	6, 113	6, 113
	*P*	0.269	0.080	0.140	0.982	0.320
Nighttime						
	Black	6.44 ± 1.65	0.11 ± 0.08	0.00 ± 0.00	0.28 ± 0.19	6.72 ± 1.66
	Blue	7.22 ± 1.85	0.39 ± 0.27	0.00 ± 0.00	0.06 ± 0.06	7.67 ± 1.84
	Brown	5.56 ± 1.44	0.00 ± 0.00	0.00 ± 0.00	0.28 ± 0.06	5.56 ± 1.44
	Green	5.00 ± 1.05	0.44 ± 0.20	0.00 ± 0.00	0.11 ± 0.08	5.50 ± 1.03
	Red	7.89 ± 1.84	0.11 ± 0.11	0.00 ± 0.00	0.56 ± 0.26	8.00 ± 1.81
	White	5.89 ± 0.80	0.00 ± 0.00	0.00 ± 0.00	0.11 ± 0.08	5.94 ± 0.80
	Yellow	6.56 ± 1.21	0.06 ± 0.06	0.00 ± 0.00	0.39 ± 0.20	6.67 ± 1.19
	*F*	0.4	0.8	—	1.1	0.4
	df	6, 114	6, 114	—	6, 114	6, 114
	*P*	0.897	0.588	—	0.394	0.886

Means within a column followed by letter were not provided as they were not significantly different (Tukey–Kramer test at *P* < 0.05).

^*a*^Includes detached segments, deep distortion, pricks, dents, merged surface impressions, scooped marks, and U-shaped impressions.

In trial 2 (July 2020), the time of exposure and model color were significantly different for the number of total impressions, but the model color × time of exposure interaction was not significantly affected (Table 1). The paired marks were only significantly different for the time of exposure. The model color significantly affected the number of scratches and other impressions (detached segments, deep distortion, pricks, dents, merged surface feeding impressions, scooped marks, and U-shaped marks). The interaction between color × time of exposure was not significant for all impression types (Table 1). To understand the effects of model colors, one ANOVA was performed by the time of exposure. The number of scratches and total impressions was significantly greater on the blue model than on the yellow model during the daytime (Table 3). There were no significant differences between the black, brown, green, red, and white models for scratches and the total number of impressions. For paired marks, cuts, and other impressions, model colors were not significantly different during the daytime. During the night, a significantly greater number of scratches were found on the red models than on the white and yellow models (Table 3). Other impression types showed significant differences between model colors, but the mean number of impressions failed to separate using the Tukey–Kramer test. Paired marks, cuts, and total impressions were not significantly affected by the model colors during the night (Table 3).

**Table 3.** ANOVA and mean (± SE) number of impressions on various colors of clay models evaluated in July 2020

Exposure		Impression type				
	Color	Paired mark	Scratch	Cut	Others^*a*^	Total
Daytime						
	Black	0.23 ± 0.11	4.70 ± 0.77ab	0.13 ± 0.06	0.60 ± 0.29	5.43 ± 0.82ab
	Blue	0.07 ± 0.05	5.73 ± 1.21a	0.13 ± 0.08	0.70 ± 0.25	6.53 ± 1.25a
	Brown	0.13 ± 0.08	2.30 ± 0.57bc	0.17 ± 0.10	0.10 ± 0.07	2.70 ± 0.63bc
	Green	0.37 ± 0.14	5.57 ± 0.92ab	0.20 ± 0.12	0.37 ± 0.13	6.43 ± 0.89a
	Red	0.10 ± 0.10	4.60 ± 0.71ab	0.30 ± 0.17	0.37 ± 0.13	5.37 ± 0.81ab
	White	0.60 ± 0.37	2.93 ± 0.61abc	0.10 ± 0.07	0.53 ± 0.18	4.03 ± 0.88abc
	Yellow	0.13 ± 0.10	1.53 ± 0.35c	0.00 ± 0.00	0.20 ± 0.07	1.83 ± 0.38c
	*F*	1.3	4.9	0.3	1.7	5.5
	df	6, 194	6, 194	6, 194	6, 194	6, 194
	*P*	0.279	<0.001	0.948	0.129	<0.001
Nighttime						
	Black	0.83 ± 0.27	4.27 ± 0.71ab	0.73 ± 0.36	0.23 ± 0.11	5.97 ± 0.81
	Blue	0.57 ± 0.22	3.97 ± 0.73ab	0.33 ± 0.24	0.60 ± 0.17	5.40 ± 0.79
	Brown	0.53 ± 0.18	3.70 ± 0.89ab	0.60 ± 0.43	0.17 ± 0.07	5.00 ± 1.00
	Green	0.80 ± 0.29	3.79 ± 0.84ab	0.87 ± 0.44	0.70 ± 0.25	5.83 ± 1.04
	Red	0.50 ± 0.20	6.07 ± 0.90a	0.53 ± 0.27	0.97 ± 0.33	8.07 ± 0.99
	White	0.60 ± 0.23	2.33 ± 0.46b	0.30 ± 0.15	0.60 ± 0.33	3.83 ± 0.72
	Yellow	1.20 ± 0.54	2.17 ± 0.58b	0.33 ± 0.30	0.23 ± 0.12	3.93 ± 1.00
	*F*	0.6	2.7	0.6	2.3	2.1
	df	6, 194	6, 194	6, 194	6, 194	6, 194
	*P*	0.725	0.017	0.764	0.038	0.055

Means within a column followed by different letters were significantly different (Tukey–Kramer test at *P* < 0.05). Where no differences were observed, no letters are included.

^*a*^Includes detached segments, deep distortion, pricks, dents, merged surface impressions, scooped marks, and U-shaped impressions.

**Fig. 5.**
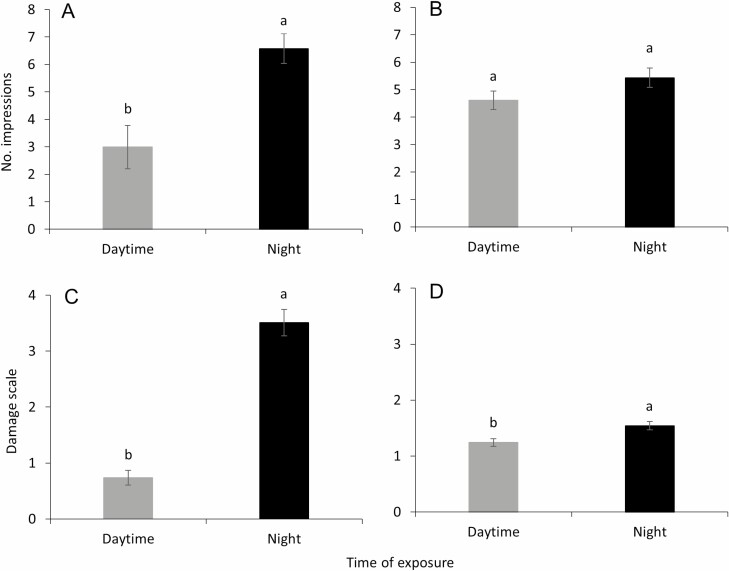
Means (± SE) total impressions recorded in daytime and night during (A) trial 1 (May 2020) and (B) trial 2 (July 2020). Means (± SE) damage scale (0–10) for daytime and night during (C) trial 1 (May 2020) and (D) trial 2 (July 2020). Bars with similar-case letters (upper or lower) are not significantly different (α = 0.05; Tukey–Kramer test).

When the effects of model color were evaluated for impressions during the daytime and night, a significantly greater number of total impressions was observed at night than during the daytime (*F* = 12.7; df = 1, 244; *P* < 0.001; Fig. 5A) during trial 1. In trial 2 (July 2020), the total number of impressions did not significantly differ (*F* = 2.6; df = 1, 409; *P* = 0.105; Fig. 5B). A similar trend was observed at the damage scale (0–10), which differed significantly between daytime and night during May (*F* = 102.8; df = 1, 244; *P* < 0.001; Fig. 5C) and July 2020 (*F* = 7.1; df = 1, 409; *P* = 0.008; Fig. 5D), with night having a significantly higher damage scale than daytime.

During the daytime in trial 1 (May 2020), paired marks were significantly correlated with scratches for the brown, green, and red models (Table 4). In the red model, cut impressions were significantly associated with scratches. The cut impression was significantly correlated with total impressions for black, brown, yellow, and white models. On blue, green, and red, a significant correlation was found between paired marks and total impression. The other impressions were significantly associated with total impressions on white models (Table 4). In July 2020, during the daytime, scratches were significantly correlated with total impressions in all the color models. The cut and paired mark impressions were significantly associated with the total number of impressions on the red and white models. Other impressions were significantly correlated with the total number of impressions on yellow models (Table 4).

**Table 4.** Pearson’s correlation between impression types by the color of the clay model during the daytime

Color		May 2020					July 2020				
		Cut	Paired	Scratch	Others	Total	Cut	Paired	Scratch	Others	Total
Black	Cut					0.99***					
	Paired										
	Scratch										0.96***
	Others										
	Total										
Blue	Cut										
	Paired					0.89***					
	Scratch										0.98***
	Others										
	Total										
Brown	Cut					0.92***					
	Paired			0.71**							
	Scratch										0.98***
	Others										
	Total										
Green	Cut										
	Paired			0.56*		0.86***					
	Scratch					0.87***					0.97***
	Others										
	Total										
Red	Cut			0.87***		0.96***					0.38*
	Paired			0.59**		0.59**					0.50*
	Scratch				0.60*	0.96***					0.96***
	Others										
	Total										
White	Cut					0.99***					0.57**
	Paired										0.74***
	Scratch										0.88***
	Others					0.99***					
	Total										
Yellow	Cut					0.97***					
	Paired										
	Scratch										0.95***
	Others										0.37*
	Total										

The notations indicate the correlation (*P < 0.05; **P < 0.01; and ***P < 0.001) between different impression types.

During the night in trial 2, the paired marks were significantly correlated with the total number of impressions in all the color treatments (Table 5). Additionally, paired marks were significantly associated with the other impressions in black models. In July 2020, a significant association between scratches and other impressions was observed in the brown, green, and yellow models. Cut impression and paired marks were significantly correlated with the red model, whereas the cut impression was significantly correlated with scratches on the yellow model. Paired marks were significantly correlated with the total number of impressions in the brown and white models. The scratches were significantly associated with the total number of impressions in all the color treatments.

**Table 5.** Pearson’s correlation between impression types by the color of clay model during nighttime

Color	May 2020						July 2020				
		Cut	Paired	Scratch	Others	Total	Cut	Paired	Scratch	Others	Total
Black	Cut										
	Paired					0.99***					
	Scratch				0.56*						0.84***
	Others										
	Total										
Blue	Cut										
	Paired					0.99***					
	Scratch										0.91***
	Others										
	Total										
Brown	Cut										
	Paired					1.00***					0.41*
	Scratch									0.42*	0.90***
	Others										
	Total										
Green	Cut										
	Paired					0.98***					
	Scratch									0.57**	0.85***
	Others										0.62**
	Total										
Red	Cut							0.41*			
	Paired					1.00***					
	Scratch										0.85***
	Others										
	Total										
White	Cut										
	Paired					1.00***				0.40*	0.45*
	Scratch										0.78***
	Others										0.70***
	Total										
Yellow	Cut								0.50**		0.68***
	Paired					1.00***					
	Scratch									0.37*	0.76***
	Others										
	Total										

The notations indicate the correlation (*P < 0.05; **P < 0.01; and ***P < 0.001) between different impression types.

### Shape and Size

In trial 1 (July 2020), the effect of shape and the size of the clay model was significant on the total number of impressions observed on clay models; however, their interaction was not significant (Table 6). The shape was significantly different on the number of paired marks, but there were no significant differences in size and shape and size interaction. The shape and size were significantly different in the number of scratches. A significant effect was found on shape and size interaction on prick impressions on clay models. When the effect of size was analyzed by shape, significantly greater numbers of scratches and total impressions were found on large models than on medium models, followed by small models (Table 7). The prick impressions were significantly greater on large-sized models than on medium- and small-sized models. For the beetle-shaped models, the large- and medium-sized models captured significantly greater impressions than the small models. The size of the beetle shape was not significantly different for any distinct impression type (Table 7).

**Table 6.** Summary of ANOVA results for the model used to find the effect of the shape, size, and their interaction on different impression types in July and August 2020

Impression	Shape			Size			Shape × size		
	F	df	P	F	df	P	F	df	P
July 2020									
Paired	13.9	1, 173	<0.001	2.7	2, 173	0.069	0.1	2, 173	0.913
Scratch	16.2	1, 173	<0.001	10.6	2, 173	<0.001	1.7	2, 173	0.178
Prick	0.0	1, 173	0.872	2.9	2, 173	0.058	3.8	2, 173	0.024
Other	0.0	1, 173	0.997	0.0	2, 173	0.992	1.0	2, 173	0.367
Total	22.1	1, 173	<0.001	12.8	2, 173	<0.001	1.3	2, 173	0.270
August 2020									
Paired	0.0	1, 173	0.960	2.9	2, 173	0.056	0.0	2, 173	0.959
Scratch	3.9	1, 173	0.049	6.0	2, 173	0.003	0.1	2, 173	0.892
Prick	0.1	1, 173	0.799	1.3	2, 173	0.288	0.9	2, 173	0.391
Other	193.8	1, 173	<0.001	128.2	2, 173	<0.001	486.0	1, 173	<0.001
Total	2.3	1, 173	0.130	7.2	2, 173	0.001	0.3	2, 173	0.769

**Table 7.** ANOVA and mean (± SE) number of impressions on various shapes of clay models evaluated in July 2020

Shape		Impression type				
	Size	Paired mark	Scratch	Prick	Others^*a*^	Total
Larvae	Large	1.60 ± 0.35	3.47 ± 0.50a	0.83 ± 0.19a	0.07 ± 0.05	5.97 ± 0.70a
	Medium	1.13 ± 0.36	1.67 ± 0.41b	0.20 ± 0.09b	0.17 ± 0.10	3.17 ± 0.55b
	Small	0.77 ± 0.22	0.60 ± 0.18c	0.20 ± 0.11b	0.10 ± 0.06	1.67 ± 0.43c
	*F*	2.1	13.5	6.7	0.5	13.7
	df	2, 78	2, 78	2, 78	2, 78	2, 78
	*P*	0.134	<0.001	0.002	0.609	<0.001
Beetle	Large	0.57 ± 0.19	0.83 ± 0.26	0.30 ± 0.13	0.20 ± 0.11	1.90 ± 0.42a
	Medium	0.47 ± 0.31	0.80 ± 0.18	0.57 ± 0.21	0.07 ± 0.07	1.90 ± 0.52a
	Small	0.23 ± 0.14	0.37 ± 0.14	0.17 ± 0.07	0.00 ± 0.00	0.76 ± 0.29b
	*F*	1.3	1.9	2.0	0.5	4.0
	df	2, 78	2, 78	2, 78	2, 78	2, 78
	*P*	0.284	0.161	0.138	0.597	0.023

Means within a column followed by different letters were significantly different (Tukey–Kramer test at *P* < 0.05). Where no differences were observed, no letters are included.

^*a*^Includes dents, merged surface impressions, elongated scratches, scooped marks.

In trial 2 (August 2020), the size was significantly different on the total number of impressions, whereas shape and shape and size interaction was not significantly different (Table 6). Paired marks were not significantly different for shape, size, or their interaction. The effect of size and shape was significantly different for the number of scratches on clay models. There were no significant effects of prick impression on shape, size, or their interaction. For other impressions, shape, size, and shape and size interactions were significantly different (Table 6). When the analysis was performed by shape to understand the effect of size, for larvae shape, there were no differences between sizes for any distinct impression type and the total number of impressions (Table 8). For beetle shape, the total number of impressions was significantly greater on the large models than on the small models. The impression types were not significantly different between sizes.

**Table 8.** Mean ± SE number of impressions on various shapes of clay models evaluated in August 2020

Shape		Impression type				
	Size	Paired	Scratch	Prick	Others^*a*^	Total
Larvae	Large	1.23 ± 0.45	2.10 ± 0.69	0.30 ± 0.17	0.37 ± 0.17	4.00 ± 0.89
	Medium	0.53 ± 0.13	1.63 ± 0.32	0.23 ± 0.09	0.13 ± 0.09	2.53 ± 0.46
	Small	0.87 ± 0.26	0.77 ± 0.27	0.36 ± 0.21	0.10 ± 0.06	2.10 ± 0.50
	*F*	1.6	3.0	0.2	1.5	2.7
	df	2, 78	2, 78	2, 78	2, 78	2, 78
	*P*	0.215	0.056	0.822	0.240	0.072
Beetle	Large	1.33 ± 0.71	1.47 ± 0.33a	0.63 ± 0.19	0.20 ± 0.10	3.63 ± 0.80a
	Medium	0.53 ± 0.18	0.87 ± 0.28ab	0.20 ± 0.09	0.13 ± 0.10	1.73 ± 0.38ab
	Small	0.77 ± 0.20	0.47 ± 0.22b	0.27 ± 0.10	0.00 ± 0.00	1.50 ± 0.34b
	*F*	0.6	2.9	2.8	0.1	4.4
	df	2, 78	2, 78	2, 78	2, 78	2, 78
	*P*	0.547	0.058	0.068	0.868	0.015

Means within a column followed by different letters were significantly different (Tukey–Kramer test at *P* < 0.05). Where no differences were observed, no letters are included.

^*a*^Includes dents, merged surface impressions, elongated scratches, scooped marks.

**Fig. 6.**
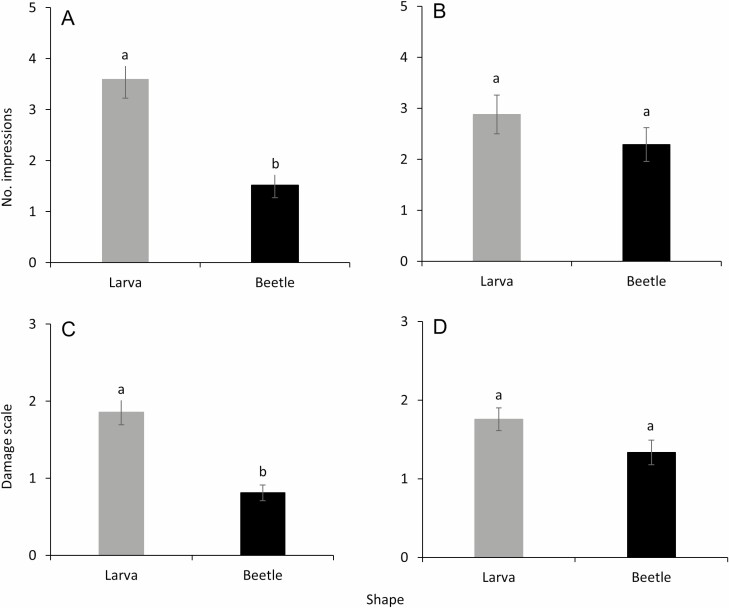
Means (± SE) total impressions recorded on larval- and beetle-shaped models (A) trial 1 (July 2020) and (B) trial 2 (August 2020). Means (± SE) damage scale (0–10) on larval and beetle-shaped models (C) trial 1 (July 2020) and (D) trial 2 (August 2020). Bars with similar-case letters (upper or lower) are not significantly different (α = 0.05; Tukey–Kramer test).

When the effects of shape were evaluated for total impressions, a significantly greater numbers of total impressions were observed on the larva-shaped models than on the beetle-shaped models (*F* = 21.9; df = 1, 177; *P* < 0.001; Fig. 6A) in trial 1 (July 2020). In trial 2 (August 2020), the total numbers of impressions did not significantly differ (*F* = 1.5; df = 1, 177; *P* = 0.216; Fig. 6B) between the larva-shaped and the beetle-shaped models. The damage scale values were significantly greater for larva-shaped models than for beetle-shaped models in July 2020 (*F* = 30.2; df = 1, 177; *P* < 0.001; Fig. 6C). In trial 2 (August 2020), the damage scale values did not significantly differ between the larva-shaped and the beetle-shaped models (*F* = 3.8; df = 1, 177; *P* = 0.053; Fig. 6D).

In the Pearson’s correlation analysis for trial 1 (July 2020), paired marks were significantly correlated with prick impression, and scratches were significantly correlated with prick impression for beetle shape (Table 9). For larvae and beetle shapes, paired marks, scratches, and prick impressions were significantly correlated with the total number of impressions. In trial 2 (August 2020), paired marks were significantly different from prick impressions for larval shape. There were significant correlations between paired marks, scratches, and prick impressions and the total number of impressions on both the caterpillar- and beetle-shaped models (Table 9).

**Table 9.** Pearson′s correlation between impression types by the shape of clay model

Shape		July 2020					August 2020				
		Paired	Scratch	Prick	Others	Total	Paired	Scratch	Prick	Others	Total
Larvae											
	Paired					0.66***					0.67***
	Scratch					0.84***					0.81***
	Prick					0.40***	0.22*				0.38***
	Others										
	Total										
Beetle											
	Paired					0.78***					0.81***
	Scratch			0.24**		0.69***					0.56***
	Prick	0.42***				0.70***					0.32**
	Others										
	Total										

The notations indicate the correlation (*P < 0.05; **P < 0.01; and ***P < 0.001) between different impression types.

## Discussion

The results showed that the clay model is an effective tool in capturing a range of arthropod-mediated impressions in turfgrass. The blue and green models had greater densities of impressions than the yellow or white models during daytime. During the nighttime, however, all colored models captured similar numbers of impressions, although a greater number of impressions were recorded during the night than during the day, implying that either densities of predators or their activity were greater during the night than during the day, and predaceous behavior was not influenced by prey color. Previously, studies showed that successful host searching and acceptance involved chemical cues from prey (Yasuda 1997, Xue et al. 2018), herbivore-induced plant volatiles (Drukker et al. 2000, Schuman and Baldwin 2016), or a combination of tactile, visual, olfactory (smell), and gustatory (taste) cues (Halpin and Rowe 2016, Duong et al. 2017, Manubay and Powell 2020, Yamazaki et al. 2020). Data also suggest that diurnal predators, especially birds, use visual cues to spot suitable prey (Zvereva et al. 2019, Yamazaki et al. 2020). Green models were used the most in past research due to their resemblance to foliage-feeding larvae and lack of warning coloration (Low et al. 2014, Sam et al. 2015, Roels et al. 2018, Khan and Joseph 2021, Long and Frank 2020).

The larval-shaped models captured more impressions than the beetle-shaped clay models, and the density of impressions increased with the increase in the size of the model. Troscianko et al. (2009) suggested that the shape of the prey subject is one of the important factors that can influence predatory interactions. Although it is unclear why predators preferred one shape over the other, it is possible that arthropod predators evolved on preying on larval stages of insects, and they are selected for traits that can recognize less mobile immature stages of arthropods. Additionally, the number of impressions increased with an increase in the size of the model in the current study. A previous study showed that the size of the body of the prey influenced the preference of ground beetle, *Pterostichus melanarius* (Illiger) (Coleoptera: Carabidae) (McKemey et al. 2001). Similarly, the size of the mandibles of different species of tiger beetles in the genus *Cicindela* was correlated with the average size of prey (Pearson and Mury 1979). Smaller prey subjects are preferred by carabids, *Nebria brevicollis* (Fabricius) and *Pterostichus madidus* (Fabricius), when compared to larger slugs (Mair and Port 2001). A previous study also showed that predator size and morphology can influence how they interact with prey. The macrocephalic morph of the ground beetle *Damaster blaptoides* Kollar, with a large head and strong jaws, prefers to crush the prey, the snail species in the genera *Acusta*, *Aegista*, *Bradybaena*, *Cochlicopa*, *Discus*, *Euhadra*, *Succinea*, *Satsuma*, *Stereophaedusa*, and *Zaptychopsis*; whereas the stenocephalic morph of the same predator species with a narrow head and weak jaws prefers to consume the soft body by inserting the head into the snail shell aperture (Konuma and Chiba 2007), suggesting that predator interactions could vary by species, and more research is warranted to understand species-specific effects on clay models. Clearly, the current study indicated that the increased size of the prey model would benefit the capture of more predatory interactions if the goal is to monitor predatory activity.

Predators leave behind unique impressions on the clay model, and some of those impressions can be used to identify the specific type of predators active in the system (Low et al. 2014, Khan and Joseph 2021). Most of the impressions found in the current study were characterized by Khan and Joseph (2021) by exposing common turfgrass arthropods to clay models in laboratory assays. Paired marks, scratches, cuts and pricks, and other impressions were the impression types observed in the current study (Fig. 3). Some of the common arthropods reported from the central Georgia turfgrass fields are *Calosoma sayi* DeJean, *Tetracha carolina* (L.), *Scarites subterraneus* Fabricius, *Harpalus pensylvanicus* De Geer, *Anisodactylus* sp., *Amara* sp., *Agonum* sp. (all Coleoptera: Carabidae), Elateridae (Coleoptera), *Sphenophorus* spp. (Coleoptera: Curculionidae), *Neocurtilla hexadactyla* (Perty) (Orthoptera: Gryllotalpidae), *Labidura riparia* (Pallas) (Dermaptera: Labiduridae), *Euborellia annulipes* (Lucas) (Dermaptera: Anisolabididae), *Solenopsis invicta* Buren (Hymenoptera: Formicidae), *Pseudopachybrachius vinctus* (Say) (Hemiptera: Rhyparochromidae), and Lycosidae (Araneae) and these arthropods interacted with clay models (Khan and Joseph 2021). Besides arthropods, avian community can cause impressions on the clay models (Low et al. 2014). Specifically, cut impressions can be caused by birds (Low et al. 2014) as well as carabids (Khan and Joseph 2021). In the current study, the clay models were placed within the grass canopy, which reduce light reflected from the surface of the model. This suggests that the incidence of avian predation is minimal in the current study. However, the hunting bird, such as European starling, *Sturnus vulgaris* L. (Passeriformes: Sturnidae), often search for prey while walking on the turfgrass (Vittum 2020) and they may locate the clay model and potentially interact with them.

More impressions were found during nighttime than during the daytime, suggesting that most of the predators present in turfgrass could be active at night. Our result is consistent with a previous study conducted in a temperate forest, where a greater level of predation was observed during the night than during the daytime (Ferrante et al. 2017). In contrast, higher predatory activity was observed on the clay models during the daytime than during the nighttime in a rainforest habitat in another study (Seifert et al. 2016). Cheng et al. (2018) showed that lower levels of predation on dark-shaded lepidopteran models than on those models placed in open habitats, suggesting that the timing of model deployment can vary by ecosystem-specific characteristics and activity behavior of prevalent prey and predator species (Ferrante et al. 2017, Hernández-Agüero et al. 2020). Noctuid pests, such as *S. frugiperda* larvae, have a nocturnal habit, and it is possible that predators in turfgrass systems have also evolved with the nocturnal habits of prey. When surveys were conducted at night on creeping bentgrass (*Agrostis stolonifera* L.), active populations of carabids and ants were documented attacking nocturnal turfgrass pest, *A. ipsilon* (Hong et al. 2011). In addition to light, other abiotic factors, such as variations in temperature, relative humidity, and precipitation, can influence predator–prey interactions (Laws 2017). The effects of abiotic factors on predator activity and interactions in clay models warrant more research to enhance the utility of clay models in turfgrass environments.

The incidence of types of impressions was not similar across various colored models. The scratch impressions were relatively lower on light-colored shades such as white- and yellow-colored models than on dark-shaded models, perhaps an issue of reduced detectability because of poor light contrast under the lighted stereomicroscope. These results are consistent with a recent study conducted in Mediterranean woodlands, where lower levels of predatory interactions were observed with lighter-shaded clay models (yellow models) than with the darker-shaded (brown- and black-colored models) (Hernández-Agüero et al. 2020). Similarly, Ferrante et al. (2017) also showed that a greater interaction events from predators on red clay models than on green clay models. Scratch impressions were associated with paired marks, and in some instances, paired marks were associated with prick impressions (Fig. 3F; Tables 4, 5, and 9). These results indicate that some of the predators make multiple impressions when they interact with models. It is also possible that impressions on clay models are generated from non-predatory origins. For example, scratch impressions can be caused by accidental crawling of adult billbugs (*Sphenophorus* spp.) on the models (Khan and Joseph 2021) or through unintentional contact with grass blades. Impressions can be generated through anthropogenic origins, such as while handling and transporting clay models. Thus, the implications of certain impressions, such as scratches, should be carefully interpreted, as knowledge of the arthropod community prevalent in a given system is essential and will complement the utility of the clay model.

To summarize, the results showed that impressions created on the clay model were not influenced by the color of the model during the night, whereas more impressions were found on the blue and green models than on the white or yellow models during the day. When the shape of the models represented lepidopteran larvae and carabid adults, more impressions were found in lepidopteran larvae-shaped models than in adult beetle-shaped models. More impressions were found on the models as the size of the models increased, regardless of shape. These results lay out characteristics of clay models to maximize the detection of predator activity in a turfgrass system. The use of the clay model tool can be enhanced to understand the relative activity of predators, which emphasizes the need for the conservation of predators for pest management and improves integrated pest management approaches in turfgrass.

## References

[CIT0001] Aslam, M., O. Nedvěd, and K. Sam. 2020. Attacks by predators on artificial cryptic and aposematic insect larvae. Entomol. Exp. Appl. 168: 184–190.

[CIT0002] Bateman, P. W., P. A. Fleming, and A. K. Wolfe. 2017. A different kind of ecological modelling: the use of clay model organisms to explore predator–prey interactions in vertebrates. J. Zool. 301: 251–262.

[CIT0003] Boecklen, W. J., C. T. Yarnes, B. A. Cook, and A. C. James. 2011. On the use of stable isotopes in trophic ecology. Annu. Rev. Ecol. Evol. Syst. 42: 411–440.

[CIT0004] Braman, S. K., A. F. Pendley, and W. Corley. 2002. Influence of commercially available wildflower mixes on beneficial arthropod abundance and predation in turfgrass. Environ. Entomol. 31: 564–572.

[CIT0005] Braman, S. K., R. R. Duncan, W. W. Hanna, and M. C. Engelke. 2003. Arthropod predator occurrence and performance of *Geocoris uliginosus* (Say) on pest-resistant and susceptible turfgrasses. Environ. Entomol. 32: 907–914.

[CIT0006] Braman, S. K., R. R. Duncan, W. W. Hanna, and M. C. Engelke. 2004. Species and cultivar influences on survival and parasitism of fall armyworm. J. Econ. Entomol. 97: 1993–1998. 15666756 10.1093/jee/97.6.1993

[CIT0007] Cabrera, G., O. M. Nuneza, J. Wiesner, and R. Jaskuła. 2019. Hunting in the rain: unusual behavior by the tiger beetle *Cylindera discreta elaphroides* (Doktouroff) (Coleoptera: Cicindelidae) in atropical forest on Cebu Island, Philippines. Coleopt. Bull. 73: 408.

[CIT0008] Cheng, W., S. Xing, Y. Chen, R. Lin, T. C. Bonebrake, and A. Nakamura. 2018. Dark butterflies camouflaged from predation in dark tropical forest understories. Ecol. Entomol. 43: 304–309.

[CIT0009] Del Toro, I., and R. R. Ribbons. 2020. No Mow May lawns have higher pollinator richness and abundances: an engaged community provides floral resources for pollinators. PeerJ. 8: e10021. 33024642 10.7717/peerj.10021 PMC7518183

[CIT0010] Denan, N., W. M. Wan Zaki, A. R. Norhisham, R. Sanusi, D. M. Nasir, F. Nobilly, A. Ashton-Butt, A. M. Lechner, and B. Azhar. 2020. Predation of potential insect pests in oil palm plantations, rubber tree plantations, and fruit orchards. Ecol. Evol. 10: 654–661. 32015833 10.1002/ece3.5856 PMC6988529

[CIT0011] Drukker, B., J. Bruin, and M. W. Sabelis. 2000. Anthocorid predators learn to associate herbivore-induced plant volatiles with presence or absence of prey. Physiol. Entomol. 25: 260–265.

[CIT0012] Duong, T. M., A. B. Gomez, and T. N. Sherratt. 2017. Response of adult dragonflies to artificial prey of different size and colour. PLoS One. 12: 1–14. 10.1371/journal.pone.0179483 PMC5491015 28662042

[CIT0013] Dupuy, M. M., and R. A. Ramirez. 2019. Consumptive and non-consumptive effects of predatory arthropods on billbug (Coleoptera: Dryophthoridae) pests in turfgrass. Biol. Control. 129: 136–147.

[CIT0014] Eickhoff, T. E., F. P. Baxendale, and T. M. Heng-Moss. 2006. Host preference of the chinch bug, *Blissus occiduus*. J. Insect Sci. 6: 7. 10.1673/1536-2442(2006)6[1:HPOTCB]2.0.CO;2 PMC2990293 19537992

[CIT0015] Eitzinger, B., N. Abrego, D. Gravel, T. Huotari, E. J. Vesterinen, and T. Roslin. 2019. Assessing changes in arthropod predator–prey interactions through DNA-based gut content analysis—variable environment, stable diet. Mol. Ecol. 28: 266–280. 30230073 10.1111/mec.14872

[CIT0016] Ferrante, M., G. Barone, M. Kiss, E. Bozóné-Borbáth, and G. L. Lövei. 2017. Ground-level predation on artificial caterpillars indicates no enemy-free time for lepidopteran larvae. Community Ecol. 18: 280–286.

[CIT0017] Gunnarsson, B., J. Wallin, and J. Klingberg. 2018. Predation by avian insectivores on caterpillars is linked to leaf damage on oak (*Quercus robur*). Oecologia. 188: 733–741. 30116876 10.1007/s00442-018-4234-z PMC6208694

[CIT0018] Halpin, C. G., and C. Rowe. 2016. The effect of distastefulness and conspicuous coloration on the post-attack rejection behaviour of predators and survival of prey. Biol. J. Linn. Soc. 120: 236–244.

[CIT0019] Hariraveendra, M., T. P. Rajesh, A. P. Unni, and P. A. Sinu. 2020. Prey-predator interaction suggests sacred groves are not functionally different from neighbouring used lands. J. Trop. Ecol. 36: 220–224.

[CIT0020] Haydu, J. J., A. W. Hodges, and C. R. Hall. 2008. Estimating the economic impact of the U.S. golf course industry: challenges and solutions. HortScience. 43: 759–763.

[CIT0075] Held, D. W., and D. A. Potter. 2012. Prospects for managing turfgrass pests with reduced chemical inputs. Annu. Rev. Entomol. 57: 329–354. 21910640 10.1146/annurev-ento-120710-100542

[CIT0021] Hernández-Agüero, J. A., V. Polo, M. García, D. Simón, I. Ruiz-Tapiador, and L. Cayuela. 2020. Effects of prey colour on bird predation: an experiment in Mediterranean woodlands. Anim. Behav. 170: 89–97.

[CIT0022] Hong, S. C., D. W. Held, and R. C. Williamson. 2011. Generalist predators and predation of black cutworm agrotis ipsilon larvae in close mown creeping bentgrass. Fla. Entomol. 94: 714–715.

[CIT0023] Howe, A., G. L. Lövei, and G. Nachman. 2009. Dummy caterpillars as a simple method to assess predation rates on invertebrates in a tropical agroecosystem. Entomol. Exp. Appl. 131: 325–329.

[CIT0024] Iverson, S. J., C. Field, W. D. Bowen, and W. Blanchard. 2004. Quantitative fatty acid signature analysis: a new method. Ecol. Monogr. 74: 211–235.

[CIT0025] Joseph, S. V., and S. K. Braman. 2009a. Influence of plant parameters on occurrence and abundance of arthropods in residential turfgrass. J. Econ. Entomol. 102: 1116–1122. 19610427 10.1603/029.102.0333

[CIT0026] Joseph, S. V., and S. K. Braman. 2009b. Predatory potential of *Geocoris* spp. and *Orius insidiosus* on fall armyworm in resistant and susceptible turf. J. Econ. Entomol. 102: 1151–1156. 19610431 10.1603/029.102.0337

[CIT0027] Joseph, S. V., and S. K. Braman. 2011. Occurrence of hymenopteran parasitoids in residential turfgrass in central Georgia. J. Entomol. Sci. 46: 112–123.

[CIT0028] Joseph, S. V., and S. K. Braman. 2016. Influence of turf taxa and insecticide type on survival of *Geocoris* spp. (Hemiptera: Geocoridae). J. Entomol. Sci. 47: 227–237.

[CIT0029] Joseph, S. V., K. Harris-Shultz, D. Jespersen, B. Vermeer, and C. Julian. 2020. Incidence and abundance of bees and wasps (Hymenoptera) in centipedegrass lawns in Georgia. J. Entomol. Sci. 55: 547–559.

[CIT0030] Kamenova, S., C. Leroux, S. E. Polin, and M. Plantegenest. 2018. Community-wide stable isotope analysis reveals two distinct trophic groups in a service-providing carabid community. Bull. Entomol. Res. 108: 130–139. 28615084 10.1017/S0007485317000542

[CIT0031] Khan, F. Z. A., and S. V. Joseph. 2021. Characterization of impressions created by turfgrass arthropods on clay models. Entomol. Exp. Appl. 169: 508–518.

[CIT0032] Konuma, J., and S. Chiba. 2007. Trade-offs between force and fit: extreme morphologies associated with feeding behavior in carabid beetles. Am. Nat. 170: 90–100. 17853994 10.1086/518182

[CIT0033] Laws, A. N . 2017. Climate change effects on predator–prey interactions. Curr. Opin. Insect Sci. 23: 28–34. 29129279 10.1016/j.cois.2017.06.010

[CIT0034] Li, J., F. Yang, Q. Wang, H. Pan, H. Yuan, and Y. Lu. 2017. Predation by generalist arthropod predators on *Apolygus lucorum* (Hemiptera: Miridae): molecular gut-content analysis and field-cage assessment. Pest Manag. Sci. 73: 628–635. 27349598 10.1002/ps.4346

[CIT0035] Long, L. C., and S. D. Frank. 2020. Risk of bird predation and defoliating insect abundance are greater in urban forest fragments than street trees. Urban Ecosyst. 23: 519–531.

[CIT0036] López, R., and D. A. Potter. 2000. Ant predation on eggs and larvae of the black cutworm (Lepidoptera: Noctuidae) and Japanese beetle (Coleoptera: Scarabaeidae) in turfgrass. Environ. Entomol. 29: 116–125.

[CIT0037] Lövei, G. L., and M. Ferrante. 2017. A review of the sentinel prey method as a way of quantifying invertebrate predation under field conditions. Insect Sci. 24: 528–542. 27686246 10.1111/1744-7917.12405

[CIT0038] Low, P. A., K. Sam, C. McArthur, M. R. C. Posa, and D. F. Hochuli. 2014. Determining predator identity from attack marks left in model caterpillars: guidelines for best practice. Entomol. Exp. Appl. 152: 120–126.

[CIT0039] Mair, J., and G. R. Port. 2001. Predation by the carabid beetles *Pterostichus madidus* and *Nebria brevicollis* is affected by size and condition of the prey slug *Deroceras reticulatum*. Agric. For. Entomol. 3: 99–106.

[CIT0040] Mansion-Vaquié, A., M. Ferrante, S. M. Cook, J. K. Pell, and G. L. Lövei. 2017. Manipulating field margins to increase predation intensity in fields of winter wheat (*Triticum aestivum*). J. Appl. Entomol. 141: 600–611.

[CIT0041] Manubay, J. A., and S. Powell. 2020. Detection of prey odours underpins dietary specialization in a Neotropical top-predator: how army ants find their ant prey. J. Anim. Ecol. 89: 1165–1174. 32097493 10.1111/1365-2656.13188

[CIT0042] McKemey, A. R., W. O. C. Symondson, D. M. Glen, and P. Brain. 2001. Effects of slug size on predation by *Pterostichus melanarius* (Coleoptera: Carabidae). Biocontrol Sci. Technol. 11: 81–91.

[CIT0043] Milesi, C., C. D. Elvidge, and R. R. Nemani. 2009. Assessing the extent of urban irrigated areas in the United States, pp. 217–236. *In* P. Thenkabail, J. G. Lyon, H. Turral, and C. Biradar (eds.), Remote sensing of global croplands for food security. CRC Press, Boca Raton, FL.

[CIT0044] Molleman, F., T. Remmel, and K. Sam. 2016. Phenology of predation on insects in a tropical forest: temporal variation in attack rate on dummy caterpillars. Biotropica. 48: 229–236.

[CIT0045] Moura, R. F., E. Tizo-Pedroso, and K. Del-Claro. 2018. Colony size, habitat structure, and prey size shape the predation ecology of a social pseudoscorpion from a tropical savanna. Behav. Ecol. Sociobiol. 72: 1–9.

[CIT0046] Nachappa, P., L. P. Guillebeau, S. K. Braman, and J. N. All. 2006. Susceptibility of twolined spittlebug (Hemiptera: Cercopidae) life stages to entomophagous arthropods in turfgrass. J. Econ. Entomol. 99: 1711–1716. 17066803 10.1603/0022-0493-99.5.1711

[CIT0047] Nair, S., S. K. Braman, and P. Raymer. 2021. Susceptibility of zoysiagrasses to the fall armyworm (Lepidoptera: Noctuidae). J. Entomol. Sci. 56: 24–31.

[CIT0048] Nason, L. D., P. K. Eason, M. M. Carreiro, A. Cherry, and J. Lawson. 2021. Caterpillar survival in the city: attack rates on model lepidopteran larvae along an urban-rural gradient show no increase in predation with increasing urban intensity. Urban Ecosyst. 24: 1–12.

[CIT0049] Oliveira-Hofman, C., V. S. Victor, L. J. Meinke, and J. A. Peterson. 2020. Molecular gut-content analysis of adult ground beetles (Coleoptera: Carabidae) provides no evidence of predation of western corn rootworm (Coleoptera: Chrysomelidae) in a Nebraska corn agroecosystem. J. Entomol. Sci. 55: 448–461.

[CIT0050] Paluh, D. J., E. K. Kenison, and R. A. Saporito. 2015. Frog or fruit? The importance of color and shape to bird predators in clay model experiments. Copeia. 103: 58–63.

[CIT0051] Pearson, D. L., and E. J. Mury. 1979. Character divergence and convergence among tiger beetles (Coleoptera: Cicindelidae). Ecology. 60: 557–566.

[CIT0052] Pena, J. C., F. Aoki-Gonçalves, W. Dáttilo, M. C. Ribeiro, and I. MacGregor-Fors. 2021. Caterpillars’ natural enemies and attack probability in an urbanization intensity gradient across a Neotropical streetscape. Ecol. Indic. 128: 107851.

[CIT0053] Pfannenstiel, R. S., and K. V. Yeargan. 2002. Identification and diel activity patterns of predators attacking *Helicoverpa zea* (Lepidoptera: Noctuidae) eggs in soybean and sweet corn. Environ. Entomol. 31: 232–241.

[CIT0054] Potter, D. A., and S. K. Braman. 1991. Ecology and management of turfgrass insects. Annu. Rev. Entomol. 36: 383–406.

[CIT0055] Remmel, T., and T. Tammaru. 2009. Size-dependent predation risk in tree-feeding insects with different colouration strategies: a field experiment. J. Anim. Ecol. 78: 973–980. 19493131 10.1111/j.1365-2656.2009.01566.x

[CIT0082] Robbins, P., and T. Birkenholtz. 2003. Turfgrass revolution: Measuring the expansion of the American lawn. Land Use Policy 20: 181–194.

[CIT0056] Roels, S. M., J. L. Porter, and C. A. Lindell. 2018. Predation pressure by birds and arthropods on herbivorous insects affected by tropical forest restoration strategy. Restor. Ecol. 26: 1203–1211.

[CIT0057] Rojas, B., P. Rautiala, and J. Mappes. 2014. Differential detectability of polymorphic warning signals under varying light environments. Behav. Processes. 109 Pt B: 164–172. 25158931 10.1016/j.beproc.2014.08.014

[CIT0058] Rößler, D. C., H. Pröhl, and S. Lötters. 2018. The future of clay model studies. BMC Zool. 3: 1–5.

[CIT0059] Rößler, D. C., S. Lötters, J. Mappes, J. K. Valkonen, M. Menin, A. P. Lima, and H. Pröhl. 2019. Sole coloration as an unusual aposematic signal in a Neotropical toad. Sci. Rep. 9: 1128. 30718568 10.1038/s41598-018-37705-1 PMC6362010

[CIT0060] Sahayaraj, K., and S. M. Fernandez. 2021. The predation behavior and the prey size preferences of *Antilochus coquebertii* (Pyrrhocoridae) against *Dysdercus koenigii* (Pyrrhocoridae). Int. J. Trop. Insect Sci. 41: 1763–1769.

[CIT0061] Sam, K., T. Remmel, and F. Molleman. 2015. Material affects attack rates on dummy caterpillars in tropical forest where arthropod predators dominate: an experiment using clay and dough dummies with green colourants on various plant species. Entomol. Exp. Appl. 157: 317–324.

[CIT0062] SAS Institute. 2012. SAS version 9.3. SAS Inst. Inc., Cary, NC.

[CIT0063] Schuman, M. C., and I. T. Baldwin. 2016. The layers of plant responses to insect herbivores. Annu. Rev. Entomol. 61: 373–394. 26651543 10.1146/annurev-ento-010715-023851

[CIT0064] Seifert, C. L., C. H. Schulze, T. C. T. Dreschke, H. Frötscher, and K. Fiedler. 2016. Day vs. night predation on artificial caterpillars in primary rainforest habitats – an experimental approach. Entomol. Exp. Appl. 158: 54–59.

[CIT0065] Singh, G . 2020. Improving integrated pest management strategies for the fall armyworm (Lepidoptera: Noctuidae) in turfgrass. M.S. thesis. University of Georgia, Georgia.

[CIT0066] Stier, J. C., K. Steinke, E. H. Ervin, F. R. Higginson, and P. E. McMaugh. 2015. Turfgrass Benefits and Issues, pp. 105–145. *In* J. C. Stier, B. P. Horgan, S. A. Bonos (eds.), Turfgrass: biology, use, and management. American Society of Agronomy, Crop Science Society of America, Soil Science Society of America, Inc., Madison, WI.

[CIT0079] Théry, M., and D. Gomez. 2010. Insect colours and visual appearance in the eyes of their predators, pp. 267–353. *In* J. Casas and S.J. Simpson (eds.), Advances in insect physiology: insect integument and colour. Academic Press, Cambridge, MA.

[CIT0067] Tillman, G., M. Toews, B. Blaauw, A. Sial, T. Cottrell, E. Talamas, D. Buntin, S. Joseph, R. Balusu, H. Fadamiro, et al. 2020. Parasitism and predation of sentinel eggs of the invasive brown marmorated stink bug, *Halyomorpha halys* (Stål) (Hemiptera: Pentatomidae), in the southeastern US. Biol. Control. 145: 104247.

[CIT0068] Troscianko, T., C. P. Benton, P. G. Lovell, D. J. Tolhurst, and Z. Pizlo. 2009. Camouflage and visual perception. Philos. Trans. R. Soc. B Biol. Sci. 364: 449–461. 10.1098/rstb.2008.0218 PMC2674079 18990671

[CIT0069] Vittum, P. J . 2020. 24. Vertebrate pests, pp. 362–369. *In* Turfgrass Insects of the United States and Canada. Cornell University Press, Ithaca, NY.

[CIT0070] Xue, H. J., J. Wei, Z. Z. Huang, W. Z. Li, and X. K. Yang. 2018. Your chemical coat tells me you are my delicacy: a predatory stink bug uses cuticular hydrocarbons to identify prey. Chemoecology. 28: 69–73.

[CIT0071] Yamazaki, Y., E. Pagani-Núñez, T. Sota, and C. R. A. Barnett. 2020. The truth is in the detail: predators attack aposematic prey with less aggression than other prey types. Biol. J. Linn. Soc. 131: 332–343.

[CIT0072] Yasuda, T . 1997. Chemical cues from *Spodoptera litura* larvae elicit prey-locating behavior by the predatory stink bug, *Eocanthecona furcellata*. Entomol. Exp. Appl. 82: 349–354.

[CIT0073] Zou, Y., J. De Kraker, F. J. J. A. Bianchi, M. D. Van Telgen, H. Xiao, and W. Van Der Werf. 2017. Video monitoring of brown planthopper predation in rice shows flaws of sentinel methods. Sci. Rep. 7: 1–9. 28211500 10.1038/srep42210 PMC5314450

[CIT0074] Zvereva, E. L., B. Castagneyrol, T. Cornelissen, A. Forsman, J. A. Hernández-Agüero, T. Klemola, L. Paolucci, V. Polo, N. Salinas, K. J. Theron, et al. 2019. Opposite latitudinal patterns for bird and arthropod predation revealed in experiments with differently colored artificial prey. Ecol. Evol. 9: 14273–14285. 31938518 10.1002/ece3.5862 PMC6953658

